# Prevalence, clinical characteristics, and management of irritable bowel syndrome in Vietnam: A scoping review

**DOI:** 10.1002/jgh3.12616

**Published:** 2021-07-21

**Authors:** Duc Trong Quach, Khoa Tien Vu, Khien Van Vu

**Affiliations:** ^1^ Department of Internal Medicine University of Medicine and Pharmacy at Hochiminh City Ho Chi Minh City Vietnam; ^2^ Department of Gastroenterology Nhan Dan Gia Dinh Hospital Ho Chi Minh City Vietnam; ^3^ Medical Affairs Ipsen Ho Chi Minh City Vietnam; ^4^ Department of Endoscopy 108 Central Hospital Hanoi Vietnam

**Keywords:** irritable bowel syndrome, management, prevalence, Vietnam

## Abstract

Irritable bowel syndrome (IBS) is one of the most common gastrointestinal disorders worldwide. Its prevalence varies significantly from country to country, largely due to heterogeneity in the available data. Recent studies show that the prevalence of IBS in Asia is on the rise. However, there are very limited data regarding its prevalence in the Vietnamese population. This review aims to offer an overview of the prevalence and clinical characteristics of IBS in the Vietnamese population; and to discuss the current management of IBS in Vietnam—taking into account the available medical resources and the local spectrum of lower gastrointestinal disorders that may mimic IBS.

## Graphical abstract

This review aims to offer an overview of the prevalence and clinical characteristics of irritable bowel syndrome (IBS) in the Vietnamese population; and to discuss the current management of IBS in Vietnam, taking into account the available medical resources and the local spectrum of lower gastrointestinal disorders that may mimic IBS.

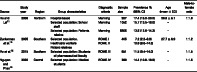

## Introduction

Although irritable bowel syndrome (IBS) is not life‐threatening, its chronic, recurrent nature has a substantial negative impact on all dimensions of quality of life (QoL).^1^, ^2^ The pooled global prevalence of IBS is about 11.2%.^3^ The prevalence varies significantly from 1.1 to 45.0% among countries across the world. This is largely due to differences in diagnostic criteria, as well as the varied distribution of IBS risk factors, such as genetics, diet, lifestyle, gastrointestinal infection, and sociocultural factors.^3^, ^4^, ^5^ Contrasting the characteristics of IBS in different populations, therefore, would shed further light on the understanding of this disorder.

Several studies in Asia have shown that the prevalence of IBS has been on the rise, especially in more affluent Asian countries.^6^, ^7^ Vietnam is a rapidly developing country in Southeast Asia, being the 15th most populous country in the world with an estimated population of 97.3 million.^8^ The Vietnamese, like many of their Asian counterparts, are increasingly adopting Westernized diets, lifestyles, and behaviors that can increase the risk of IBS.^4^, ^9^ The proportion of the Vietnamese population affected by IBS could, therefore, be significantly high, and the incidence might be on the rise. Surprisingly, there are very limited data in the English literature regarding the prevalence and characteristics of IBS in the Vietnamese population. Moreover, the available data are very out of date, with the latest study having been published about 15 years ago.^10^ This positions Vietnam as a country with largely unknown information about IBS. This review aims (i) to offer an overview on the prevalence and clinical characteristics of IBS in Vietnam, based on an extensive search of studies published either in English or Vietnamese; and (ii) to discuss current IBS management in Vietnam—taking into account the available medical resources and the local spectrum of lower functional gastrointestinal disorders that may mimic IBS.

## Methods

We searched PubMed, Embase, Google Scholar, Data Integration Center of the Vietnamese Ministry of Health, and the Vietnamese Inter‐Library System from January 1995 to August 2020 for studies that were in line with our scope. In addition, further articles were identified from relevant references of the retrieved articles. The search was restricted to articles in Vietnamese and English.

## Prevalence and clinical characteristics of IBS in Vietnamese

### Prevalence

There have been very few studies assessing the prevalence of IBS in the Vietnamese population during the last 25 years. These studies have used narrow population samples such as university students, employees at institutions, or those attending screening health check‐ups (Table 1). The prevalence of IBS was found to vary significantly, depending on the diagnostic criteria being used. Applying Rome I and Rome II criteria to the same sample, a local study reported a significant difference in the prevalence of IBS (7.2 and 10.9%, respectively).^10^ Similar findings have also been reported in previous studies in other populations.^3^, ^7^ In general, the prevalence of IBS tends to decrease when the stricter Rome IV criteria for abdominal pain are applied, since many patients with an IBS diagnosis according to the previous criteria are now classified as having other functional lower gastrointestinal disorders.^14^ Similar trends were reported in a recent systematic review on the global prevalence of IBS according to Rome IV criteria.^5^ A recent local study showed that the prevalence of IBS according to Rome IV criteria was significantly higher compared with that reported by previous local studies that used the older Rome criteria.^13^ However, this study involved medical students, who typically have a high level of education and a more stressful daily life compared with the general population. Considering these characteristics are well‐known risk factors for IBS,^15^, ^16^ the prevalence may have been overestimated; thus, community‐based studies are needed.

**Table 1:** Prevalence of irritable bowel syndrome (IBS), age and male‐to‐female ratio among Vietnamese patients with IBS

Source	Study year	Region	Group characteristics	Diagnostic criteria	Sample size	Prevalence % (95% CI)	Age (mean ± SD)	Male‐to‐female ratio
Ha and Lai^11^	2005	Northern	Hospital‐based	Manning	927	17.4 (15.0–20.0)	36.6 ± 5.1	1:1.6
			Selected population: School staff	Manning	1052	15.7 (13.5–18.0)	–	
			Selected population: Patients relative	Manning	5955	13.5 (12.6–14.3)	–	
Zuckerman *et al*.^10^	2005	Southern	Selected population. Healthcare workers Patient relatives	Rome I	405	7.2 (2.2–8.9)	27.7 ± 6.9	1:1.2
				Rome II		10.9 (8.0–14.3)		
Vo *et al*.^12^	2015	Southern	Selected population: Students of pharmaceutical faculty	Rome III	851	11.5 (9.4–14.0)	–	1:1.5
Nguyen and Phan^13^	2020	Central	Selected population: Medical students	Rome IV	299	14.4 (10.6–18.9)	–	1:1.5

CI, confidence interval.

### Age and gender distribution

A study conducted among patients attending screening health check‐ups reported the mean ± SD age of patients with IBS to be 38.6 ± 5.1 years (range 16–70 years).^11^ Another study conducted among healthcare workers and inpatient relatives reported a mean age of 27.7 ± 6.9 years (range 17–57 years).^10^ Two other studies were conducted among university students within an age range of 18–24 years. However, the age of IBS patients in these selective samples might not mirror that of IBS patients in the general population.^12^, ^13^ In conclusion, IBS is more prevalent among the Vietnamese population in their 20s–30s, which is similar to that reported in previous studies conducted across Asia and other parts of the world.^3^, ^6^

Regarding gender distribution, Vietnamese females are more likely to be affected by IBS compared with Vietnamese males, irrespective of the diagnostic criteria being used (Table 1). A female predominance was reported in all Vietnamese studies, but the female‐to‐male ratio was not as high as that reported in the West.^17^, ^18^ Previous studies in other Asian populations have reported heterogeneous findings.^6^

### Distribution of IBS subtypes

IBS is classified into four subtypes: IBS with predominant constipation (IBS‐C), IBS with predominant diarrhea (IBS‐D), IBS with mixed bowel habits (IBS‐M), and unclassified IBS (IBS‐U).^19^ Globally, IBS‐C and IBS‐D tend to be the predominant subtypes with equal distribution. In a meta‐analysis on the global prevalence of IBS, 23 studies reported the predominant IBS subtype, of which 14 reported subtypes as IBS‐C, IBS‐D, and IBS‐M with a prevalence of 35.0, 40.0, and 23.0%, respectively.^3^ The other nine studies also reported IBS‐U, with a more evenly distributed prevalence across the four subtypes. A recent systemic review reported that IBS‐M was the most common subtype with the Rome III criteria, while IBS‐D was the most common subtype with the Rome IV criteria.^5^ The prevalence of IBS subtypes has been reported to be variable among the Asian population.^7^ There have been seven studies reporting IBS subtypes in Vietnamese, which consistently showed that IBS‐D was the most common subtype, followed by IBS‐M and IBS‐C. This distribution was consistent regardless of the used diagnostic criteria and study year (Table 2).

**Table 2:** Distribution of reported subtypes in Vietnamese patients with irritable bowel syndrome (IBS)

						IBS subtype			
Source	Study year	Region	Group characteristics	Diagnostic criteria	Sample size	D	C	M	U
Ha and Lai^11^	1997	Northern	Hospital‐based	Manning	927	67.1	21.1	0.1	10.1
			Selected population (school staff)	Manning	1052	64.8	24.2	6.0	5.0
Pham^20^	2003	Northern	Hospital‐based	Rome II	158	30.3	25.9	43.6	18.3
Vo *et al*.^12^	2015	Southern	Selected population (students of pharmaceutical faculty)	Rome III	851	31.8	26.1	23.9	18.2
Nguyen and Nguyen^21^	2016	Northern	Hospital‐based	Rome III	191	50.8	24.1	23.0	2.1
Quach *et al*.^22^	2018	Southern	Hospital‐based	Rome III	404	50.0	6.4	42.6	1.0
Doan *et al*.^23^	2019	Southern	Hospital‐based	Rome III	181	63.5	13.8	19.9	2.8
Nguyen and Phan^13^	2020	Central	Selected population (medical students)	Rome IV	299	32.6	11.6	44.2	11.6

C, constipation‐predominant IBS subtype; D, diarrhea‐predominant IBS subtype; M, mixed‐type IBS subtype; U, unidentified IBS subtype.

### Risk factors and overlap of IBS with other functional gastrointestinal disorders

There have been only two studies looking at risk factors for IBS in the Vietnamese population.^12^, ^13^ Socioeconomic status, gender, solitary lifestyle, and smoking were not identified as risk factors for IBS in these studies. Consumption of canned food at least once a week was associated with IBS (odds ratio [OR]: 2.03, 95% confidence interval [CI]: 1.10–3.76).^12^ Anxiety and depression were also associated with IBS (OR: 1.92, 95% CI: 1.21–3.01; and OR: 2.23, 95% CI: 1.02–4.85, respectively).^12^, ^13^ The severity of stress was also associated with the risk of IBS. Compared to patients with mild anxiety, subjects with moderate or severe anxiety had a higher risk of IBS (OR: 2.11, 95% CI: 1.08–4.12).^12^ On the other hand, regular physical exercise was reported to be associated with a reduced risk of IBS (OR: 0.57, 95% CI: 0.35–0.93).^13^ Although postinfection IBS is a recognized cause of IBS, and acute gastroenteritis is common, there are limited data on patients in Vietnam and other Asian countries.^24^

The overlap between IBS and other functional lower gastrointestinal disorders in the Vietnamese population remains unknown, but an overlap between IBS and functional upper gastrointestinal disorders is very common. Dyspepsia and gastroesophageal reflux disease are reported more commonly among patients with IBS (OR: 2.87, 95% CI: 1.84–4.50; and OR: 2.81, 95% CI: 1.70–4.65, respectively).^12^ In an ongoing project to investigate the overlap of functional gastrointestinal disorders in Vietnam, we found that 14.0% (29/207) of patients with functional dyspepsia and 13.5% (31/230) of patients with gastroesophageal reflux disease also had IBS diagnosed according to Rome III criteria (Dr Quach DT, pers. comm., 2020).

### Medical‐care‐seeking behavior

There was only one study reporting the consultation rate among patients with IBS in Vietnam. However, this study, which reported that about 20.7% of patients with IBS‐type symptoms had seen a physician for their bowel complaints, was conducted more than 15 years ago and included a limited number of patients.^10^ Many Asian countries have been observing higher consultation rates, especially in more affluent cities.^6^ The economic situation of Vietnam has significantly improved over the last two decades. Consequently, we expect that the consultation rate should be much higher than before.

### QoL of Vietnamese patients with IBS

A recent review showed that there have been five studies addressing this aspect in Asia, including two from China, two from South Korea, and one from Singapore.^2^ The common instruments used to measure QoL were the Short‐Form‐36 and IBS‐specific QoL questionnaire. All these studies showed that IBS had a significantly negative impact on QoL.

There was only one study reporting the QoL of Vietnamese patients with IBS.^23^ This study recruited 181 IBS patients with a female‐to‐male ratio of 1.3 and a mean ± SD age of 43.0 ± 12.9 years. The proportion of IBS‐D, IBS‐C, IBS‐M, and IBS‐U patients was 63.5, 13.8, 19.9, and 2.8%, respectively. In this study, the overall mean ± SD IBS‐QoL score was 75.8 ± 12.6. The lowest scores were for food avoidance (56.9 ± 22.8) and interference with everyday activities (70.5 ± 18.5). This study has similar findings to studies in other Asian populations that used similar measurement. In China, the overall mean ± SD IBS‐QoL score was 71.7 ± 18.5, and the lowest score was for food avoidance (53.7 ± 26.9).^25^ The other low subscores were for dysphoria (65.0 ± 24.4) and health concern (67.4 ± 22.7). In South Korea, the overall mean ± SD IBS‐QoL score was 74.3 ± 18.4, and the lowest scores were for health concern (64.2 ± 21.7) and food avoidance (67.9 ± 25.2).^26^ In these two studies, female sex, educational level, anxiety, and depression were negatively related with lower QoL. A study conducted among Vietnamese patients with IBS found that none of these factors, but carbonated drinks, were associated with a lower QoL.^23^

## Diagnosis

The Vietnamese Association of Gastroenterology has not yet developed local guidelines for the diagnosis and management of IBS. Currently, Rome IV is the most widespread and highly advocated guidance in Vietnam.^19^ As the stricter criteria for abdominal pain are applied for IBS diagnosis in Rome IV, many patients with an IBS diagnosis according to the older Rome criteria are re‐classified as functional diarrhea and functional constipation. Similar finding has been recently reported in Malaysia, a neighboring country.^27^ The WGO global guidelines and the Asian consensus suggest that in patients with concerning features (i.e. age of onset >50 years, family history of colorectal cancer [CRC], blood in stools, unexplained weight loss, loss of appetite, nocturnal symptoms, fever, anemia, abdominal mass, and ascites), there should be prompt initiation of investigations—including colonoscopy.^28^, ^29^ However, it is important to note that CRC is among the top five cancers in Vietnam.^30^ In addition, the incidence of early‐onset CRC (i.e. ≤50‐year‐old) in the Vietnamese population is significantly high. In one study involving 400 consecutive patients diagnosed with advanced CRC conducted during 2009–2011, the proportion of patients with early‐onset CRC was 28.0%, with 11.0% of patients being under the age of 40 years.^31^ Local data, therefore, may suggest a lower threshold for the age onset. Notably, alarming features according to the WGO global guidelines were presented by only 22.3% of Vietnamese patients with early‐onset CRC.^22^ A previous study in Vietnamese patients with IBS showed that the Asia‐Pacific colorectal screening (APCS) score was a useful adjunctive tool to identify those with priority for colonoscopy (Table 3).^22^, ^33^

**Table 3:** Asia‐Pacific colorectal screening score^32^

Risk factor	Criterion	Points
Age (years)	<50	0
	50–69	2
	≥70	3
Gender	Female	0
	Male	1
Family history of colorectal cancer in a first degree relative	Absent	0
	Present	2
Smoking	Never	0
	Current or past	1

A score of 0–1 defines average risk, 2–3 moderate risk, and 4–7 high risk.

Celiac disease and inflammatory bowel diseases (IBD) are among the common differential diagnosis in patients presenting with IBS symptoms. However, the two diseases are still uncommon in Vietnam. A local study investigating the prevalence of serological markers of celiac disease in children in Hanoi found that the IgA antitransglutaminase antibodies test was positive in only 1% and that antiendomysial antibodies were all negative.^34^ The incidence and prevalence of IBD have increased rapidly in Asia.^35^ There have been increasing number of case series on IBD in Vietnam recently. C‐reactive protein (CRP) and fecal calprotectin measurement are useful to rule out IBD, but erythrocyte sedimentation rate and fecal lactoferrin have been shown to have little clinical utility.^36^ At a CRP level of ≤0.5 or fecal calprotectin level of ≤40 μg/g, there was a ≤1% probability of having IBD. The sensitivity and specificity of fecal calprotectin were both higher than those of CRP for IBD diagnosis (81 and 87% *versus* 73 and 78%, respectively).^37^ However, CRP assessment is much less expensive and more widely available compared with fecal calprotectin measurement in Vietnam.

## Treatment

Very few rigorous clinical trials have been conducted on treatments for IBS in the Vietnamese population. The trials conducted thus far have mainly used herbal medicines and have been published in local journals in Vietnamese. The typical limitations of these studies were unconvincing diagnostic criteria, small sample sizes, absence of a control arm, and heterogeneity of used herbal medicines. Therefore, this section is mostly a review of evidence‐based management of IBS with special attention to the available options in Vietnam.

### Diet

#### Low‐fermentable oligosaccharides, disaccharides, monosaccharides, and polyol diets

The role of diets that contain low fermentable oligosaccharides, disaccharides, monosaccharides, and polyols (FODMAPs) has recently attracted a lot of interest in the management of IBS. FODMAPs are poorly digested and/or mal‐absorbed, which exerts a high osmotic effect and draws water into the small bowel lumen. When reaching the colon, they are fermented by intestinal bacteria to short‐chain fatty acids, gas, and water. Consequently, FODMAPs yield gas and fluid changes within the gut, contributing to the common symptoms of IBS such as pain, bloating, distension, and diarrhea. The recent Asian consensus on irritable bowel syndrome suggests that a low‐FODMAP diet could be helpful in patients with IBS in Asia, but further studies in Asian populations are required.^29^ The success of this dietary approach greatly depends on detailed knowledge about the FODMAP composition of popular food items. In addition, the diet should be strictly guided by trained dieticians, in order to avoid nutritional deficits and misinterpretation of outcomes. Although a few major high‐FODMAP sources in Vietnamese cuisine have been identified (Table 4), the FODMAP composition of many Vietnamese food items in a typical meal has not been fully explored. Therefore, a low‐FODMAP diet is currently not commonly applied in routine practice.

**Table 4:** Fermentable oligosaccharides, disaccharides, monosaccharides, and polyol (FODMAP) composition of popular foods and drinks in Vietnamese cuisine^38^

Type	Low FODMAP	High FODMAP
Common cereals and grains	Rice (white or brown), rice noodles, rice crackers (unflavored), sweet plain biscuits	Wheat bread, wheat noodles, rice cakes (flavored), rye bread
Vegetables, tofu, legumes, and nuts	Vegetables: broccoli, cabbage, bok choy, spinach, seaweed, okra, gailan Condiments: chili, chives, galangal, ginger, spring onion Legumes and nuts: radish, bean sprouts, cucumber, yam, green beans, sunflower seeds, sesame seeds, cassava, tofu	Vegetables: mushrooms, snow peas, karela Condiments: spring onion (white part), onion, garlic, shallots Legumes and nuts: soyabeans, split peas, kidney beans, broad beans, pistachio, mung beans, sweet corn, cashew, taro, almond
Fruits	Grapes, orange, pineapple, dragon fruit, banana, passion fruit, durian, coconut, rambutan, persimmon, longan, lychee, guava, cantaloupe, strawberries, starfruit	Watermelon, apple, sugar banana (ripe), grapefruit, nashi pear, mango, custard apple, dried fruit
Dairy products and milk substitutes	Coconut milk, cottage cheese, soya milk (from protein), lactose‐free yoghurt, lactose‐free milk	Milk (sweetened condensed, cow's, and evaporated milk), cream, soya milk (from bean), yoghurt
Commonly used herbs and spices	Soy sauce, fish sauce, sweet and source sauce, shrimp paste, coriander, paprika, cinnamon, five‐spice powder, cumin, pandan leaves	Wasabi powder

#### Traditional IBS diet

This diet was recommended by the National Institute for Health and Care Excellence (NICE) and the recent British Society of Gastroenterology (BSG) guidelines.^39^, ^40^ Patients with IBS are advised to eat frequent, small‐sized meals and to take time to eat. Heavy meals, meals with a high intake of fat, insoluble fiber, carbonated beverages, gas‐producing foods, spicy food, coffee, artificial sweeteners, and alcohol should be avoided. In addition, patients with IBS‐D should avoid artificial sweeteners, which are found in sugar‐free sweets, chewing gum, and soft drinks. Fiber intake should be reviewed and adjusted according to the symptoms. The benefits of fiber supplements seem to be limited to soluble fiber.^41^ Fiber intake should be increased slowly to avoid abdominal bloating and gas. A multicenter, parallel, single‐blind study in Sweden showed that the traditional IBS diet reduces IBS symptoms, as well as a low‐FODMAP diet.^42^ However, another randomized controlled study reported that the low‐FODMAP diet led to significantly greater improvement in individual IBS symptoms, particularly pain and bloating, compared with the modified NICE diet.^43^ Therefore, traditional IBS diet should be applied as the first‐line diet treatment in daily practice in Vietnam, but low‐FODMAP diet may be considered as the second‐line treatment whenever it is applicable. It is also important to note that intake of canned foods and carbonated beverages is linked to exacerbation of IBS symptoms in Vietnamese.^12^, ^23^

### Lifestyle modification

A recent systematic review found that exercise had significant benefits in patients with IBS regarding gastrointestinal symptoms, QoL, depression, and anxiety.^44^ The most popular exercise intervention in the study was yoga, which lasted 4–12 weeks. The recent BSG guidelines strongly recommend that all patients with IBS be advised to take regular exercise as there is some evidence that this can be beneficial, particularly for patients with constipation.^40^ The NICE guidelines suggest encouraging patients with IBS to relax and better organize their work, to reduce stress.^39^

### Probiotics

Probiotics are live microorganisms that confer health benefits on the host when they are administered in adequate amounts.^45^ As gut dysbiosis has been reported in patients with IBS and could be partly responsible for IBS symptoms,^46^ a plausible management approach is to use probiotics to alter the microbiome, so as to relieve symptoms. In fact, many meta‐analyses have shown that probiotics could deliver beneficial effects in patients with IBS symptoms with a similar safety profile to placebo.^47^, ^48^, ^49^ However, these studies could not reach definitive conclusions about the efficacy of probiotics in IBS, mainly due to the significant variability in species, strains, combinations, and doses of probiotics used in the original studies. The recent BSG guidelines recommend that probiotics, as a group, could be effective for global symptoms and abdominal pain in IBS (weak recommendation, very low evidence), but it is not possible to recommend a specific species or strain.^40^ The recent clinical guideline of American College of Gastroenterology suggests against probiotics for the treatment of global IBS symptoms (conditional recommendation; very low quality of evidence).^50^ Interestingly, a recently published meta‐analysis found that there were differences regarding geographic location, with good efficacy noted in Eastern Asia but unclear benefit in North America, Europe, South Africa, and West Asia.^48^ If patients want to try probiotics, they should be advised to take the dose recommended by the manufacturer for at least 4 weeks and up to 12 weeks given that the appropriate species, strain, and the extra cost have been well considered while monitoring the effect, and to discontinue them if there is no improvement in symptoms.^39^, ^40^ The safety profile of probiotics is generally good.^47^, ^48^

In Vietnam, there are currently two available probiotics that showed good evidence in IBS according to the updated guidelines of the World Gastroenterology Organization^45^: *Lactobacillus plantarum* 299V (DSM 9843) (10 billion colony‐forming units [CFUs] once a day) and *Saccharomyces boulardii* CNCM I‐745 (5 × 10^9^ CFUs/capsule or 250 mg twice daily). The first was registered as a food supplement and the latter as a prescription drug. In a randomized, double‐blind, placebo‐controlled multicenter trial in Korea, a regimen of *S. boulardii* at 2 × 10^11^ live cells once a day for 4 weeks was found to improve IBS‐QoL better than placebo.^51^ However, the treatment was not superior for bowel frequency and stool consistency in patients with diarrhea‐predominant IBS‐D or IBS‐M.^51^, ^52^ *L. plantarum* 299v (DSM 9843) has been reported to provide effective symptom relief at least in two randomized control trials in IBS patients. In one study, the severity and frequency of abdominal pain, bloating, and feeling of incomplete emptying were significantly improved with *L. plantarum* 299v at 10 billion CFUs daily administered for 4 weeks, compared with placebo. Moreover, the percentage of patients and investigators who rated the efficacy of the treatment as good or excellent was significantly higher in the LP299V group compared with the placebo group.^53^ In the other study, which compared *L. plantarum* 299v 20 billion CFUs daily with placebo over a 4‐week treatment period, abdominal pain was improved in both groups, but flatulence was more rapidly and significantly improved in the treatment group *versus* the placebo group. In addition, patients in the treatment group maintained better overall gastrointestinal function than those in the control group after 12 months.^54^

### Pharmaceutical management

#### Abdominal pain

Dietary and lifestyle modifications, including probiotics as food supplements, as mentioned earlier, may be beneficial for managing abdominal pain. However, some patients with severe symptoms may require additional treatment. A recent network meta‐analysis showed that antispasmodics, peppermint oil, and tricyclic antidepressants (TCAs) were more efficacious than placebo for improving abdominal pain after 4–12 weeks of treatment.^55^ The number needed to treat (NNT) and the number needed to harm (NNH) of drugs used for abdominal pain in IBS are presented in Table 5. Enteric‐coated peppermint oil capsules are not available in Vietnam.

**Table 5:** Common drug options for irritable bowel syndrome management

Symptom	Drug	NNT	NNH	Common adverse events	Reference
Abdominal pain	Antispasmodics	5	17	Some have anticholinergic side effects	Ford *et al*.^56^
	Peppermint oil	4	125	Heartburn, dry mouth, belching, peppermint taste, rash, dizziness, headache	Alammar *et al*.^57^
	Tricyclic antidepressant	4	8	Fatigue, drowsiness, urinary retention, dry mouth	Ford *et al*.^58^
Constipation	Polyethylene glycol	5	16	Abdominal pain, diarrhea, bloating, gas, dizziness	Chapman *et al*.^59^
	Lubiprostone	12	10	Diarrhea, nausea, gas, vomiting, dry mouth	Ford *et al*.^60^
	Linaclotide	6	7	Diarrhea, bloating, gas, abdominal pain or discomfort	Ford *et al*.^60^
Diarrhea	Smectite	NA	NA	Constipation, vomiting	Chang *et al*.^61^
	Loperamide	NA	NA	Constipation, dizziness, drowsiness, tiredness	Lavo *et al*.^62^
	Rifaximin	10	8971	Similar to placebo	Pimentel *et al*.^63^
	Ramoxetron	6	8	Constipation, headache, rash, itching, flushing	Fukudo *et al*.^64^

NA, not available; NNH, number needed to harm; NNT, number needed to treat.

##### Antispasmodics

Drugs in this class reduce contraction of the gastrointestinal smooth muscles, which causes abdominal cramps in patients with IBS. The second Asian consensus recommends using drugs in this class when abdominal pain is the predominant symptom.^29^ In Asia, there are many more options for antispasmodics compared with other parts of the world.^65^ However, head‐to‐head trials among antispasmodics are very limited. A double‐blind randomized control trial in Taiwan comparing otilonium bromide 40 mg three times a day and mebeverine 100 mg three times a day over 8 weeks showed that the two drugs had comparable efficacy for alleviating abdominal pain, flatulence, and bloating.^66^ Some antispasmodics have anticholinergic side effects and should be used with caution in patients with glaucoma and benign prostatic hyperplasia. The most common adverse events were dry mouth, dizziness, and blurred vision, but none of the trials reported any serious adverse events.^56^

##### Central neuromodulators

The exact mechanism of action of these drugs has not entirely been understood, but they might act on the pathways between gut and brain related to IBS. Both TCAs and selective serotonin reuptake inhibitors have comparative effectiveness for global IBS symptoms with an NNT and NNH of 4 and 8, respectively.^58^ However, only TCAs were found to be effective for abdominal pain management with an NNT of 4 (Table 5). The most commonly recommended TCAs in IBS are desipramine (25–10 mg every bedtime) and amitriptyline (10–50 mg every bedtime), but only the latter is available in Vietnam.^19^ As drowsiness is a common adverse event, the drug should be started at a low dose before bedtime and stepped up weekly, as needed. In addition, constipation is a common side effect of TCAs; hence TCAs should be used with caution in patients with IBS‐C. These drugs would be most suitable for patients with IBS‐D or IBS with sleep disturbances.

#### Constipation

The NICE guidelines recommend using laxatives for treating IBS‐C and advising patients on how to adjust the dose according to the clinical response.^39^ There is little evidence of laxatives in IBS‐C beyond PEG. A randomized control trial comparing PEG 3350 plus electrolytes *versus* placebo found that spontaneous complete bowel movements, stool consistency, and severity of straining were all significantly improved after 4 weeks in the treatment arm compared with the control arm.^59^ The severity score for abdominal pain, however, was not significantly different between the two arms. The NNT and NTH were 5 and 16, respectively (Table 5). Lactulose is a non‐absorbed disaccharide and should be avoided, as it may cause bloating.^39^ Stimulant laxatives could cause cramps and should not be considered as first‐line treatment in IBS‐C. In Vietnam, PEG 3350 is not available. In daily practice, we found that PEG 4000 is also a good option for the treatment of constipation in patients with IBS‐C. In fact, a double‐blind, randomized, parallel‐group trial in patients with chronic constipation found that PEG3350 and PEG 4000 were both well tolerated and had similar efficacy for stool consistency and date of occurrence of first spontaneous bowel movement.^67^

The NICE guidelines state that secretagogues are effective in IBS‐C with high evidence.^39^ Linaclotide, a guanylate cyclase C activator, and lubiprostone, a chloride channel activator, have been studied in Asian populations and are also considered effective treatments with high evidence for IBS‐C.^29^ These drugs, however, are currently not available in Vietnam. In our experience, most Vietnamese patients with IBS‐C respond to PEG 4000 10 g (1 sachet per day). In situations with no response, increasing the dose to 10 g PEG 4000 twice a day and combining with short‐term stimulant laxatives such as bisacodyl 5–10 mg at bedtime is usually enough. Stimulant laxatives should be used as rescue therapy if patients have no bowel movement for three consecutive days.

#### Diarrhea

The anti‐diarrheal medications commonly studied in the treatment of IBS‐D are loperamide and smectite. The former is an opioid agonist, while the latter is a natural adsorbent clay compound. Evidence for loperamide in IBS is weak, based on data from two small trials.^62^, ^68^ According to the second Asian consensus and the NICE guidelines in 2019, these drugs could be considered first‐line options, given the limited availability of drugs for IBS‐D management in many countries.^29^, ^39^ Self‐titration of loperamide dose and administration in a single nightly dose were reported to be safe and efficient in IBS‐D patients, to improve urgency, stool frequency, and stool consistency.^62^, ^68^ Smectite also has limited efficacy for diarrhea in patients with IBS‐D, but it could improve abdominal pain and bloating.^61^

There is high evidence for rifaximin, a nonabsorbable antibiotic, in the management of IBS without constipation.^63^ The rationale is to reduce gut dysbiosis, which has been described in IBS. The drug is prescribed at a dose of 550 mg, three times a day for 2 weeks. Not only stool consistency but also the global IBS symptoms of bloating and abdominal pain are significantly improved during the first 4 weeks after treatment. The disadvantage of this treatment is its high cost and short‐term efficacy, which may require repeated treatment.

A few 5‐HT3 antagonists (e.g. alosetron, ramosetron, and ondansetron) are effective in IBS‐D with high evidence.^29^ Alosetron is not available in Asia. Ramosetron was developed in Japan and has demonstrated good efficacy in global IBS symptoms, abdominal pain, and stool consistency.^64^ It is currently approved for use in only some Asian countries (Japan, Thailand, and Korea), but not yet in Vietnam.

#### Other treatments

Currently, there is very limited evidence to support the use of herbal medicines in the management of IBS in Vietnam. Regarding psychological intervention, several therapies have been reported to be more efficacious than control interventions. However, the most compelling evidence was for cognitive behavioral therapy and hypnotherapy and gut‐directed hypnotherapy.^69^ These interventions could be rescue treatments for patients whose symptoms do not respond to other treatments.^39^ However, they are currently not locally available.

In conclusion, IBS is reportedly quite prevalent in the Vietnamese population, but further population‐based studies are needed to determine the true prevalence in this population. As with IBS patients elsewhere, Vietnamese patients with IBS have a low QoL—especially given the need for food avoidance and interference with daily activities. Currently, the traditional IBS diet is more applicable compared with the low‐FODMAP diet in Vietnam. Common pharmaceutical options are locally available for individualizing management based on the predominant symptoms, quality of evidence, and patient preferences.
